# *Complement receptor 2* downregulation is associated with mortality in *Staphylococcus aureus sepsis* in both mice and humans

**DOI:** 10.3389/fimmu.2026.1724049

**Published:** 2026-07-09

**Authors:** Pradeep Kumar Kopparapu, Meghshree Deshmukh, Santhilal Subhash, Majd Mohammad, Zhicheng Hu, Anders Jarneborn, Muhammad Arif, Marcela Pekna, Lars Ljungström, Gunnar Jacobsson, Ola Grimsholm, Tao Jin

**Affiliations:** 1 Department of Rheumatology and Inflammation Research, Institute of Medicine, The Sahlgrenska Academy, University of Gothenburg, Gothenburg, Sweden; 2 Cold Spring Harbor Laboratory, Cold Spring Harbor, NY, United States; 3 Department of Biosciences and Bioengineering, Indian Institute of Technology Jammu, Jammu, Jammu and Kashmir, India; 4 Center for Clinical Laboratories, The Affiliated Hospital of Guizhou Medical University, Guiyang, China; 5 Department of Rheumatology, Sahlgrenska University Hospital, Gothenburg, Sweden; 6 Department of Molecular and Clinical Medicine, Institute of Medicine, The Sahlgrenska Academy, University of Gothenburg, Gothenburg, Sweden; 7 Science for Life Laboratory, University of Gothenburg, Gothenburg, Sweden; 8 Center for Brain Repair and Rehabilitation, Department of Clinical Neuroscience and Rehabilitation, Institute of Neuroscience and Physiology, The Sahlgrenska Academy, University of Gothenburg, Gothenburg, Sweden; 9 Department of Infectious Diseases, Skaraborg Hospital, Skövde, Sweden; 10 Institute of Pathophysiology and Allergy Research, Center for Pathophysiology, Infectiology and Immunology, Medical University of Vienna, Vienna, Austria

**Keywords:** B cells, biomarkers, *complement receptor 2*, mortality, rna sequencing, sepsis

## Abstract

Although sepsis is a leading cause of global mortality, the lack of reliable biomarkers hinders early risk stratification. Such biomarkers would facilitate timely and precise therapeutic interventions. Using a murine model of *Staphylococcus aureus* (*S. aureus*) sepsis, we performed transcriptomic analysis and identified numerous genes that distinguished survivors from fatal cases. The top 16 candidate genes were further evaluated in patients with severe invasive bacterial infections. Among these, *complement receptor 2* (*CR2*) was significantly downregulated in deceased patients compared with survivors and healthy controls. The extent of *CR2* downregulation was dependent on the administered dose of *S. aureus.* Time-course experiments conducted in infected mice showed progressive B-cell depletion and *CR2* downregulation, correlating with disease severity. Importantly, *CR2* downregulation was independent of complement factor 3, TLR2, and tumor necrosis (TNF)-α, but was reversed in infections with *S. aureus* strains that lacked sortase A and B, and restored by antibiotic treatment of the infected mice. Loss of *CR2* expression may reflect B-cell activation followed by differentiation into plasmablasts and redistribution to secondary lymphoid organs. The present study suggests that *CR2* downregulation is associated with severe infection and mortality in *S. aureus* sepsis and may represent a candidate biomarker of host immune dysregulation that warrants further validation in larger clinical cohorts.

## Introduction

Sepsis is a life-threatening syndrome caused by a dysregulated host response to infection, leading to organ dysfunction and high mortality (1). *Staphylococcus aureus* (*S. aureus*) is a major cause of sepsis (2), particularly in hospital-acquired infections (3). This pathogen employs a diverse array of virulence factors to evade immune defenses, enabling both localized and systemic infections (4). Despite advances in antimicrobial therapy and critical care, sepsis remains a major global healthcare challenge (2), with mortality rates of 10%–20% overall and up to 80% in septic shock (5, 6). Identifying biomarkers that reflect disease severity and host immune responses remains an important unmet clinical need (7), given that such markers may help guide monitoring and therapeutic decision-making.

Sepsis involves a dynamic interplay of proinflammatory and immunosuppressive responses, driven by interactions between the pathogen-associated molecular patterns (PAMPs) of invading microorganisms and host pattern recognition receptors, leading to activation of both innate and adaptive immunity (8, 9). The innate and adaptive immunity systems are tightly interconnected, with complement pathways acting as a crucial bridge. The complement system, a central component of innate immunity, is frequently activated during sepsis, thereby amplifying inflammation and contributing to tissue injury (10). Dysregulation of complement pathways has been implicated in impaired immune responses (11, 12).

Complement receptor 2 (CR2), primarily expressed on B cells with the highest levels observed in marginal zone B cells, binds the degradation products of complement component C3, such as iC3b, C3d, and C3dg (13), and plays a key role in B-cell maturation, activation, and memory formation (14). B cells, which are central to antibody production and immune regulation, are profoundly affected in sepsis. Severe infections, including those caused by *S. aureus*, are often accompanied by depletion of circulating B cells, a change has been associated with poor clinical outcomes (15–18). The numbers of specific B-cell subsets, such as immature/transitional B cells, resting memory B cells, and antibody-secreting cells, are significantly reduced in infected non-survivors (15, 19), with losses driven by intrinsic and extrinsic apoptosis, as well as pyroptosis (20). Experimental evidence shows that CR2 deficiency increases mortality in the cecal ligation and puncture model of acute septic polymicrobial peritonitis, suggesting a protective role for CR2 (21).

Clinical assessment of sepsis severity is hindered by patient heterogeneities in relation to age, sex, infecting pathogen, comorbidities, treatments received, and time from symptom onset to emergency care. To address these challenges, we adopted a translational approach, beginning with a standardized murine model and thereafter extending to human cohorts. We conducted RNA sequencing of samples from mice that were infected with defined doses of *S. aureus* for specified disease durations, in order to identify molecular signatures that distinguished survivors from non-survivors. Subsequently, we validated these signatures in patients with sepsis, to assess clinical relevance. Thus, we observed early CR2 downregulation in association with severe infection and mortality and defined outcome-related alterations in *CR2* expression and B-cell populations that occur during infection.

## Materials and methods

### Bacterial strains

*S. aureus* wild-type Newman (22) and the Newman Δ*srtAB* strain that lacks Sortase A/B expression (38) were cultured separately on horse blood agar plates for 24 h and stored as previously described (23). Newman Δ*srtAB* strain and *S. aureus* wild-type Newman have been used in studies investigating the role of sortase in septic arthritis (24, 25). The bacterial solutions were thawed, washed with sterile PBS, and adjusted to the required concentration before each experiment.

### Ethics

All experiments were conducted according to the guidelines of the Swedish Research Council and local ethical regulations, as outlined by Swedish Board of Agriculture’s recommendations for animal experiments. This study was approved by the regional Animal Ethics Committee with ethical permit number 38-2016, and the patient study was approved by the Ethical Review Board of Gothenburg with ethical permit number 376-11. Written informed consent was obtained from all volunteers before any study-related procedures were conducted.

### Mice

Female NMRI and C57Bl/6 wild-type mice of both sexes, aged 6–9 weeks, were purchased from Envigo (Venray, The Netherlands) and Charles River Laboratories (Sulzfeld, Germany), respectively. TLR2-deficient B6.129-Tlr2tm1Kir/J (TLR2^−/−^) mice of both sexes were purchased from The Jackson Laboratory (Bar Harbor, ME, USA). C3-deficient (C3^−/−^) mice were backcrossed to the C57BL/6 genetic background for 10 generations, and both sexes were used. All mice were housed in the Department of Rheumatology and Inflammation Research animal facility and the Experimental Biomedicine (EBM) unit at the University of Gothenburg. Mice were housed under standard temperature and light conditions and were fed laboratory chow and water *ad libitum*.

### *In vivo* mice experiments

#### Identification of transcriptional signatures associated with infection outcome

The experimental setup is illustrated in **Supplementary Figure 1**. Female NMRI mice (n = 12) were intravenously infected with 200 μL of the Newman WT strain (2 × 10^7^ CFU per mouse). In this model, clinical signs of sepsis typically appear between Day 3 and Day 5 post-infection. To capture early molecular changes during infection, blood samples were collected on Day 2 post-infection. Mortality was monitored daily until Day 10, when all the mice were sacrificed. Prior to blood and organ collection, mice were anesthetized by intraperitoneal injection of a ketamine (2.5 mg/kg) and detomidine hydrochloride (0.16 mg/kg) mixture prepared in 200 μL of sterile 0.9% NaCl solution. ‘Survivors’ were defined as mice alive on Day 10 after infection, whereas ‘non-survivors’ were defined as mice that either died or reached predefined humane endpoints requiring euthanasia before Day 10. No additional clinical criteria were applied for this classification. Blood samples from Days 0 and 2 were used for RNA sequencing. Based on survival outcomes, mice were retrospectively stratified into two groups (survivors and non-survivors), and transcriptomic analyses were performed to identify gene expression patterns associated with infection outcome.

To prioritize candidates for further validation, we selected the top 16 genes showing the strongest associations with survival status. These candidate genes were subsequently evaluated in patient samples. Among the 16 genes examined, only CR2 demonstrated a significant and consistent difference across all three groups: healthy controls, survivors, and non-survivors. After observing reduced *CR2* expression in association with severe infection in humans, we conducted an additional seven animal experiments to elucidate the potential mechanisms contributing to early *CR2* downregulation in sepsis. Unless otherwise stated, the blood samples obtained in those experiments were collected on Day 2 post-infection and investigators were blinded during outcome assessment and data analyses.

#### Dose-dependent CR2 expression

To assess whether the level of *CR2* expression correlates with administered bacterial dose, female NMRI mice were infected i.v. with the *S. aureus* Newman strain at either a low dose (1 × 10^5^ CFU/mouse, n = 5) or a lethal dose (1 × 10^8^ CFU/mouse, n = 5). Healthy control mice (n = 5) received PBS i.v.

#### Time-dependent CR2 expression

To examine temporal changes in *CR2* expression, female NMRI mice were infected i.v. with the *S. aureus* Newman strain (1 × 10^8^ CFU/mouse) and groups were sacrificed at 4 h (n = 5), 24 h (n = 5), and 48 h (n = 5) post-infection and the blood samples were collected.

#### Role of TNF in CR2 downregulation

To investigate the role of TNF, female NMRI mice (n = 6/group) were treated with etanercept (Enbrel; Wyeth Europa) at 500 μg/mouse 24 h before infection, and they continued receiving etanercept at the same dose daily until sacrifice at 48 h post-infection. Etanercept effectively inhibited biological activity of mouse TNF (26, 27). Mice were infected i.v. with the Newman strain (1 × 10^8^ CFU/mouse). Healthy controls included PBS-injected mice (n = 5) and etanercept -treated mice (n = 3) without infection. Blood was collected at sacrifice for FACS analysis.

#### Role of TLR2 in CR2 downregulation

C57BL/6 wild-type mice (n = 5) and congenic TLR2^-^/^-^ mice (n = 6), including both sexes, were infected i.v. with Newman strain (3.6 × 10^7^ CFU/mouse). Male and female mice were evenly distributed between the infected and control groups. After 48 h, the mice were sacrificed and blood was collected for FACS analysis. Controls included PBS-injected C57BL/6 wild-type (n = 5) and TLR2^-^/^-^ (n = 4) mice.

#### Role of C3 in CR2 expression

C57BL/6 wild-type (n = 5) and congenic C3^-^/^-^ mice (n = 4), including both sexes, were infected i.v. with Newman strain (1.3 × 10^7^ CFU/mouse). Male and female mice were evenly distributed between the infected and control groups. After 48 h, mice were sacrificed and blood was collected for FACS analysis. Controls included PBS-injected wild-type (n = 5) and C3^-^/^-^ (n = 4) mice.

#### Role of S. aureus sortase A/B in CR2 expression

Female NMRI mice were infected i.v. with either WT Newman (n = 5) or the Newman Δsortase A/B mutant (n = 5) at 1 × 10^8^ CFU/mouse. After 48 h, the mice were sacrificed and blood was collected for FACS analysis. PBS-injected mice (n = 5) served as healthy controls.

#### CR2 as a marker of treatment response

To evaluate CR2 as a potential treatment-response marker, female NMRI mice (n = 5/group) were infected i.v. with Newman strain (1 × 10^8^ CFU/mouse), and then treated at 12 h with cloxacillin (100 mg/mouse, twice daily) until sacrifice at 48 h post-infection. Healthy controls (n = 5) received PBS i.v. Blood was collected for FACS analysis.

### Flow cytometry and antibodies

Blood samples were collected from NMRI, C57BL/6 wild-type, TLR2^-^/^-^, and C3^-^/^-^ mice into EDTA tubes. Erythrocytes were lysed using RBC Lysis Buffer (eBioscience; Invitrogen, Waltham, MA, USA). Cells were stained with the following fluorochrome-conjugated antibodies: phycoerythrin (PE)-conjugated anti-CD19 (BioLegend, San Diego, CA, USA); APC-Cyanine7–conjugated anti-CD21 (BioLegend); PerCP-Cyanine5.5–conjugated anti-CD23 (BioLegend); fluorescein isothiocyanate (FITC)-conjugated anti-CD69 (Invitrogen); and allophycocyanin (APC)-conjugated anti-CD93 (Invitrogen). Fixable Viability Dye e506 (Invitrogen) was included to discriminate live cells from dead cells. Samples were acquired on a BD FACSLyric flow cytometer (BD Biosciences, Franklin Lakes, NJ, USA), and data were analyzed using the FlowJo ver. 10.8 software (BD Biosciences).

### RNA extractions and RNA sequencing

RNA samples were extracted from the blood and organs of mice using miRNeasy Mini Kit (QIAGEN, Hilden, Germany) according to the manufacturer’s instructions. The quantity and quality of the isolated RNA samples were determined using the NanoDrop 2000 spectrophotometer (Thermo Fisher Scientific, Waltham, USA), Qubit^®^ RNA HS Assay Kit (Invitrogen) and 2200 TapeStation Automated Electrophoresis System (Agilent Technologies). The samples had RNA integrity numbers (RIN) between 2.2 and 8.8, with no significant differences between groups. Because ribosomal RNA depletion libraries were used, samples with lower RNA integrity numbers were retained for transcriptomic analysis. Although RIN values ranged from 2.2 to 8.8, all samples met predefined sequencing quality criteria and generated libraries with acceptable read depth, GC content, and alignment rates. Importantly, adjustment for RIN in the differential expression analyses produced highly concordant results (>85–90% overlap of DEGs), and neither correlation heatmaps nor PCA analyses showed clustering by RIN, indicating that the observed transcriptomic patterns were driven by biological differences rather than RNA quality.

Ribosomal RNA was removed, and sequencing libraries were prepared using the TruSeq Stranded Total RNA Sample Preparation Kit with Ribo-Zero Gold (Illumina, San Diego, CA, USA), following the manufacturer’s instructions. Construction of libraries was accomplished using the TruSeq Stranded Total RNA Sample Preparation Guide 15031048 Rev. E (Illumina), using 0.5–1.0 µg of total RNA. The Novaseq 6000 platform was used, and 100-bp paired-end reads were generated by Clinical Genomics (Gothenburg, Sweden).

16 candidate genes for human validation were prioritized based on statistical significance (adjusted p<0.01) and strong differential expression (|log_2_ fold change| ≥ 2), and biological relevance to immune pathways implicated in sepsis.

### Transcriptome analysis of healthy and *S. aureus-*infected mouse samples

Paired-end, strand-specific (fr-first strand) sequencing reads from this study were aligned to the Ensembl *Mus musculus* reference genome (GRCm39) (28) using HISAT2 (v2.2.1) (29). The resulting alignment files were sorted and indexed with SAMtools (v1.5) (30), and gene-level quantification was performed with featureCounts from the Subread package (v2.0.0) (31) using paired-end and strand-specific parameters (−p -s 2 -B --minOverlap 10 -Q 30 --ignoreDup). Gene annotation was obtained from GENCODE (32), corresponding to the GRCm39 assembly. Differential expression analysis between biological conditions was conducted in R (v4.1.1) using the DESeq2 Bioconductor package with median-of ratios normalization and multiple-testing correction was applied using the Benjamini–Hochberg false discovery rate procedure (33). Genes with adjusted p-values < 0.01 and an absolute log_2_ fold-change ≥ 1 were considered significantly differentially expressed. These genes were subjected to functional enrichment analysis against the KEGG and Gene Ontology databases using GeneSCF (v1.1-p2) (34), with significant pathways defined by p-values < 0.05.

### cDNA synthesis, and quantitative RT-PCR

Total cDNA synthesis was performed using the Superscript III First-Strand Synthesis Supermix Kit (Invitrogen) according to the manufacturer’s protocol. The expression levels of mouse and human genes were analyzed using Power SYBR Green gene expression assays (Applied Biosystems, Warrington, UK). The β-actin genes of mice (*Actb*), and humans (*ACTB*) were used as the internal controls, respectively. The predesign primers are readily available (KiCqStart SYBR Green; Merck, Darmstadt, Germany). The differences in expression were calculated using the ΔΔCt method. The primer sequences are listed in the **Supplementary Table 2**.

### *In vitro* stimulation of splenocytes

Splenocytes were extracted from healthy NMRI mice (n=6) under sterile conditions (35). Briefly, the mouse spleens were removed, mashed aseptically, and passed through a 70-µm-pore cell strainer (Fisher Scientific). Erythrocytes were depleted by lysis in 0.83% ammonium chloride. The B cells were then isolated by negative selection using the EasySep Mouse Pan-B Cell Isolation Kit (STEMCELL Technologies, Vancouver, Canada), according to the manufacturer’s instructions. The purity of B cell population is higher than 90%. The isolated B cells were washed and suspended in Iscove’s complete medium (10% fetal calf serum, 2 mM L-glutamine, 5 × 10–^5^ M mercaptoethanol, 50 µg/mL gentamicin) to a density of 2 × 10^6^ cells/mL.

B-splenocytes were stimulated with TSST-1 (100 ng/mL), heat-killed *S. aureus* bacteria (1 × 10^7^ CFU/mL), PAM3CSK4 (20 ng/mL), and lipopolysaccharide (LPS) (1 µg/mL), while Iscove’s medium with cells was used as the unstimulated control. The cells were incubated (at 37 °C in 5% CO_2_) for 3, 5, and 7 days, for the FACS analysis. Cell viability was assessed using Fixable Viability Dye eFluor 506, and only viable cells were included in the flow cytometric analyses. The vast majority of cells remained viable throughout the experiments. To ensure reproducibility, the *in vitro* stimulation experiments were performed in two independent biological experiments using splenocytes isolated from separate mice, with a total of five mice included. Data from these independent experiments were pooled for analysis.

### Patients and healthy controls

Twenty-nine patients who presented at the emergency department at Skaraborg Hospital in western Sweden and who were subsequently diagnosed with invasive bacterial infection were prospectively included in the study (**Supplementary Table 3**). Invasive bacterial infection was defined as the isolation of bacteria from a normally sterile site, including blood, synovial fluid, cerebrospinal fluid, pleural fluid, bone, or deep-seated abscesses. Bacteraemia was defined as the presence of at least one positive blood culture. All positive blood cultures were reviewed and classified as true infections based on clinical history, physical examination findings, clinical course, microbiological results, and response to treatment. The final diagnosis was established using a combination of clinical, radiological, and microbiological information.

Blood samples were collected at the time of presentation to the emergency department, before initiation of antibiotic therapy; therefore, no patients had received antibiotic treatment prior to sampling. Patients with sepsis fulfilled the clinical diagnostic criteria used by the treating physicians at the time of hospital admission. Information regarding major comorbidities was not systematically collected and was therefore not available for analysis.

The mean age of the patients was 71 years; 52% were men and 48% were women. Mortality was assessed within 28 days, as short-term lethality more accurately reflects the outcome of acute infectious disease. Non-survivors were significantly older than survivors (mean age: 80 vs. 67 years), whereas no gender differences were observed between the groups. For comparison, five age- and sex-matched healthy individuals were recruited as controls. All samples were stored at –70 °C until further analysis by RT-PCR or ELISA.

Data from patients with sepsis and healthy controls were obtained from the Gene Expression Omnibus (GEO) repository with accession number GSE154918 (36). Raw count data, Transcripts per Million (TPM) values, and the corresponding gene annotation file were downloaded directly from GEO. Gene identifiers were mapped to gene symbols using the provided annotation file. Differential gene expression analysis between sepsis patients and healthy controls was performed on raw count data using DESeq2, while TPM values were used for data visualization. For correlation analyses, TPM expression levels of selected genes were compared with HLA-DMB expression across all samples using Spearman’s rank correlation. Sample metadata were used to annotate clinical groups, and scatter plots were generated with samples colored according to group assignment. Linear regression lines were included for visualization purposes, and each plot was annotated with the sample size, Spearman correlation coefficient, and nominal p-value. No additional sample inclusion or exclusion criteria were applied beyond those reported in the original GSE154918 study. Since all samples originated from a single publicly available dataset processed using a standardized protocol, no additional batch correction was performed.

### Statistical analysis

All statistical analyses were performed using GraphPad Prism ver. 9 (GraphPad Software, La Jolla, CA, USA). Statistical differences were assessed using the Mann-Whitney *U*-test and data are presented as the mean with standard deviation (SD); * *p* < 0.05; ** *p* < 0.01.

## Results

### The *CR2* expression is altered in association with lethal *S. aureus* infection in mice

**Figure 1:**
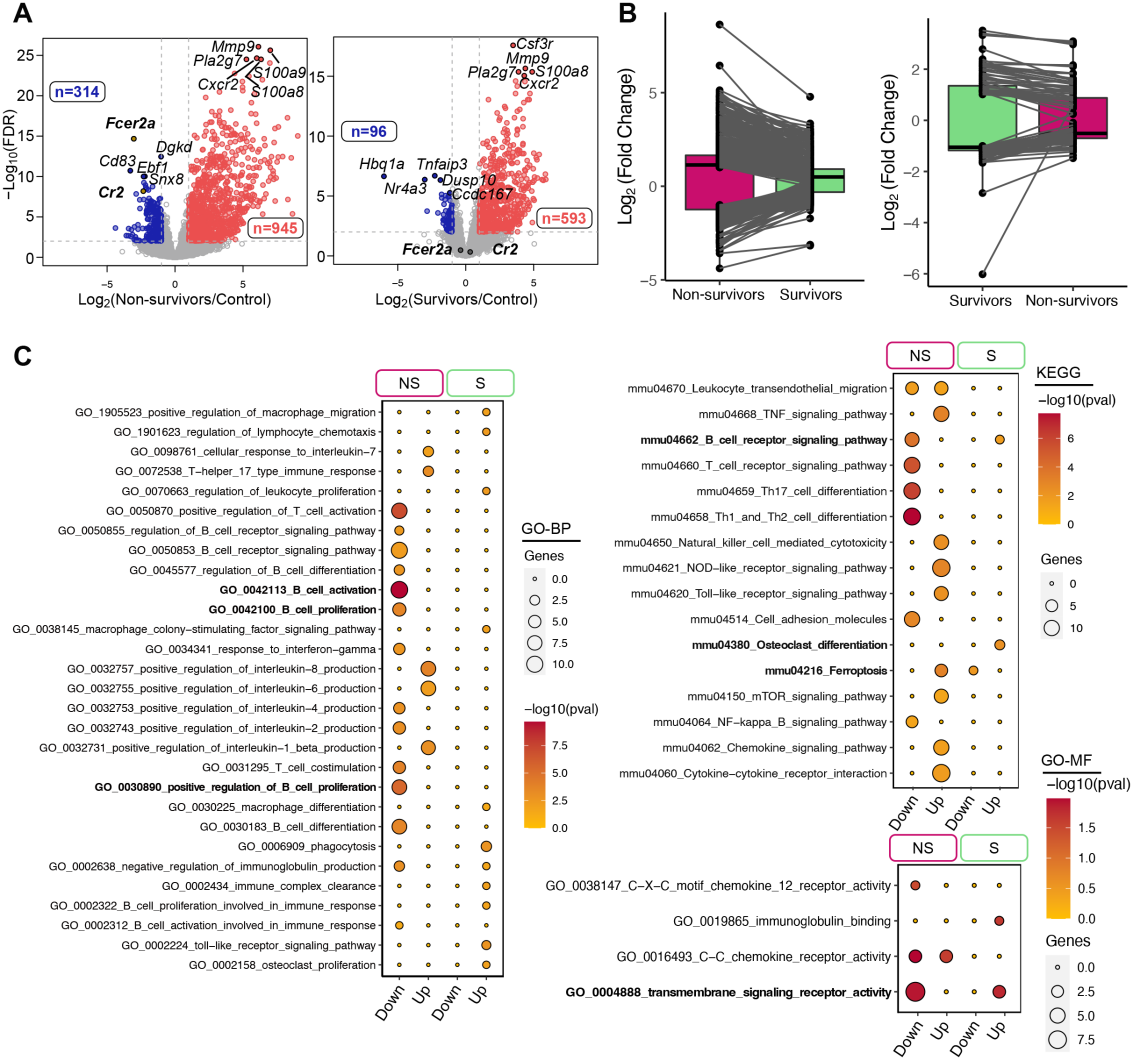
Transcriptomics analysis of surviving and non-surviving mice following *S. aureus* infection. NMRI mice (n = 12) were infected intravenously with the *Staphylococcus aureus* (*S. aureus*) Newman strain (2 ×10^7^ CFU/mouse). Blood samples were collected at baseline (Day 0) and on Day 2 post-infection, prior to the onset of clinical sepsis. Mice were monitored twice daily until Day 10, during which four mice died (non-survivors) and eight survived (survivors). RNA sequencing was performed on blood samples from healthy controls (n = 4), non-survivors (NS, n = 4), and survivors (S, n = 8). Bioinformatic analyses were performed to compare transcriptional profiles between non-survivors, survivors, and healthy controls in order to identify gene expression patterns associated with infection outcome. **(A)** Volcano plots showing differentially expressed genes between the Day 0 controls and the Day 2 samples from non-survivors (left) or survivors (S) (right). The horizontal dotted line indicate a false discovery rate (FDR) cut-off of 0.05, and the vertical dotted lines indicate a log_2_-fold change cut-off of ±1. The red and blue colors indicate upregulated and downregulated genes, respectively. **(B)** Boxplots showing genes specifically deregulated in non-survivors (NS) and their expression in survivors (S) (left), and vice versa (right). **(C)** Gene Ontology (GO-BP, Biological Process; GO-MF, Molecular Function) and KEGG pathways enriched among genes differentially regulated in non-survivors (NS) and survivors (S). The circle size indicates the number of differentially expressed genes, and the color gradient indicates the level of statistical significance.

To identify molecular signatures associated with infection outcome in *S. aureus-*induced sepsis, we analyzed blood samples from *S. aureus*-infected mice and a healthy control group (**Supplementary Figure 1**). We performed transcriptomics/total RNA profiling of the deceased/non-survivor group (n=4) and survivor group (n=8) after infection with *S. aureus*, as well as a healthy/control group (n=4). In total, 945 genes were upregulated and 314 were downregulated in the non-survivors compared with the controls, whereas 593 genes were upregulated and 96 downregulated in the survivors compared with the controls. Direct comparison of transcriptional profiles between survivors and non-survivors further highlighted a subset of genes specifically altered in non-survivors, including CR2 and other B-cell related genes (**Supplementary Figure 2**). Among these genes, the complement receptor (*CR2*) and *Fc Fragment of IgE Receptor II* (*Fcer2a/CD23*) genes were significantly downregulated in non-survivors, although their levels were unchanged in survivors relative to controls (**Figure 1A**). Moreover, the genes that were specifically deregulated in non-survivors did not show significant changes in surviving mice. In contrast, several genes that were specifically deregulated in the survivors showed similar altered expression levels in the non-survivors. This indicates that the transcriptional changes is specifically associated with the non-survivor-phenotype (**Figure 1B**). Pathway enrichment analysis revealed significant reductions in B-cell activation, proliferation, and receptor signaling in the non-survivor group, consistent with impaired B-cell responses during severe infection (**Figure 1C**). In addition, the osteoclast differentiation pathway was significantly downregulated in non-survivors compared with survivors (**Figure 1C**). These findings identify non-survivor-associated transcriptional changes, particularly within the B-cell related pathways during severe *S. aureus* sepsis in mice.

### Reduced *CR2* expression is associated with mortality in patients with severe bacterial infections

**Figure 2:**
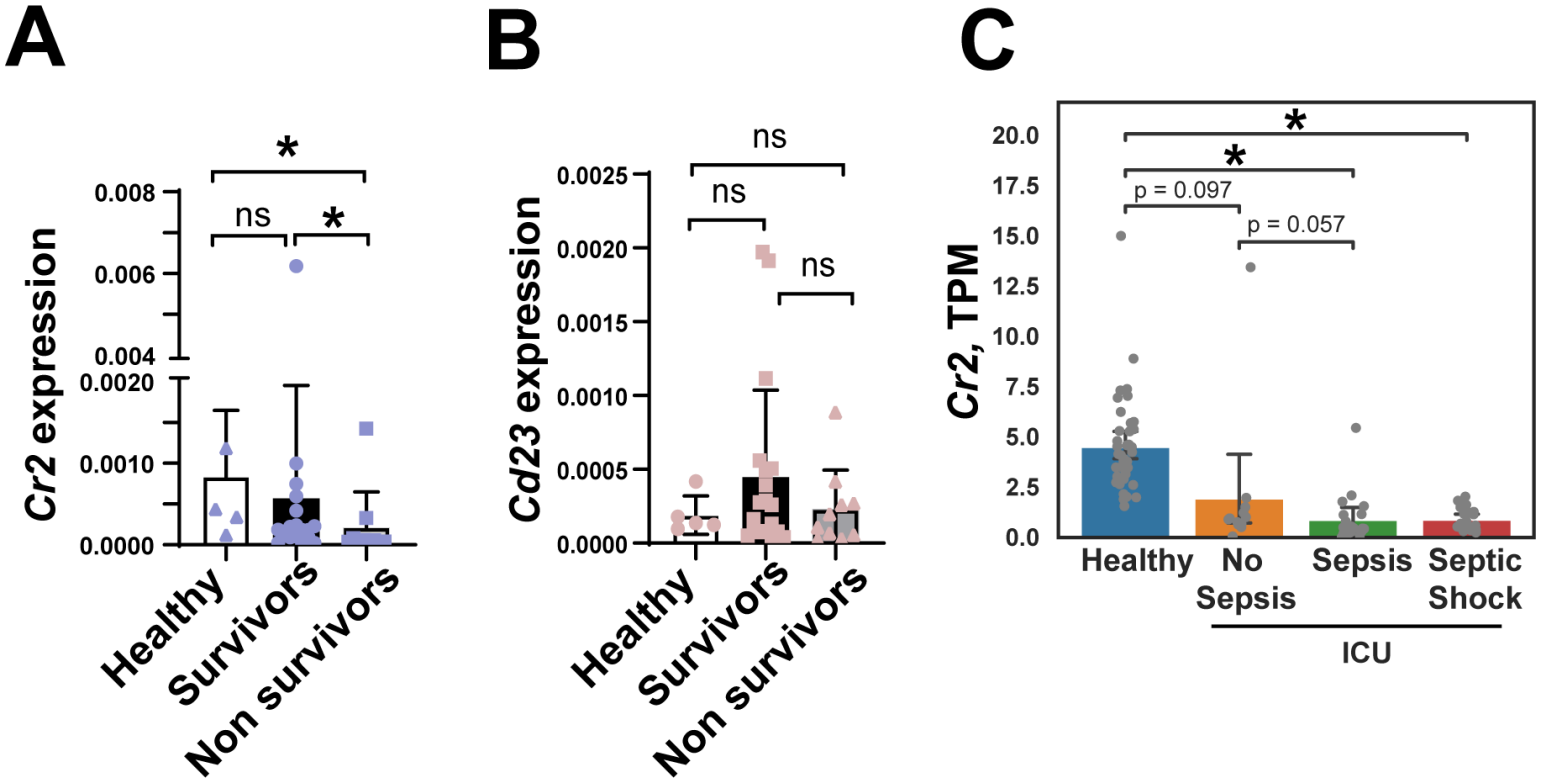
Reduced *CR2* expression in blood samples from non-surviving patients with severe bacterial infections. Patterns of mRNA expression for *CR2* **(A)** and *CD23* **(B)** in patients with bacterial infections. Statistical analyses were performed using the Mann-Whitney *U*-test and the data are presented as the mean ± SEM. **(C)** The *CR2* expression levels were further analyzed using an independent publicly available dataset (GSE154918), comprising healthy controls and ICU patients with various stages of sepsis. Raw counts and TPM data were obtained directly from the repository, and differential CR2 expression between the groups was assessed using DESeq2. *p < 0.05, **p < 0.01; n.s. = not significant.

From the mouse transcriptomics data, we prioritized the candidate genes showing strongest differential expression and biological relevance and selected 16 genes for evaluation in a cohort of patients with severe bacterial infections, including both survivors and non-survivors (**Supplementary Table 1**). Among these candidates, only CR2 demonstrated a significant and consistent difference across all three groups (Healthy controls, survivors, and non-survivors) and was the only gene significantly associated with mortality (**Supplementary Table 1**). For this reason, we focused subsequent mechanistic studies on CR2. Notably, *CR2* expression was significantly downregulated in deceased patients compared with both age-matched healthy controls and surviving patients (**Figure 2A**). Since CR2 is primarily expressed in B cells, we also examined the expression of another B-cell marker, *Fcer2a/CD23*, to assess whether this change reflected a general reduction in B- cell markers. *CD23* levels did not differ significantly among the groups (**Figure 2B**).

To further explore this observation, we analyzed an independent, publicly available database (GSE154918), containing transcriptomic data from healthy controls and intensive care unit (ICU) patients at various sepsis stages (36). Consistent with our findings, patients with sepsis and those experiencing septic shock showed significantly lower levels of *CR2* expression than the healthy controls and patients with no sepsis (**Figure 2C**).

As among these candidates, only CR2 showed a significant difference between survivors and non-survivors in the human samples. For this reason, we focused subsequent mechanistic studies on CR2.

### Infective dose-dependent and temporal regulation of CR2 expression in sepsis

**Figure 3:**
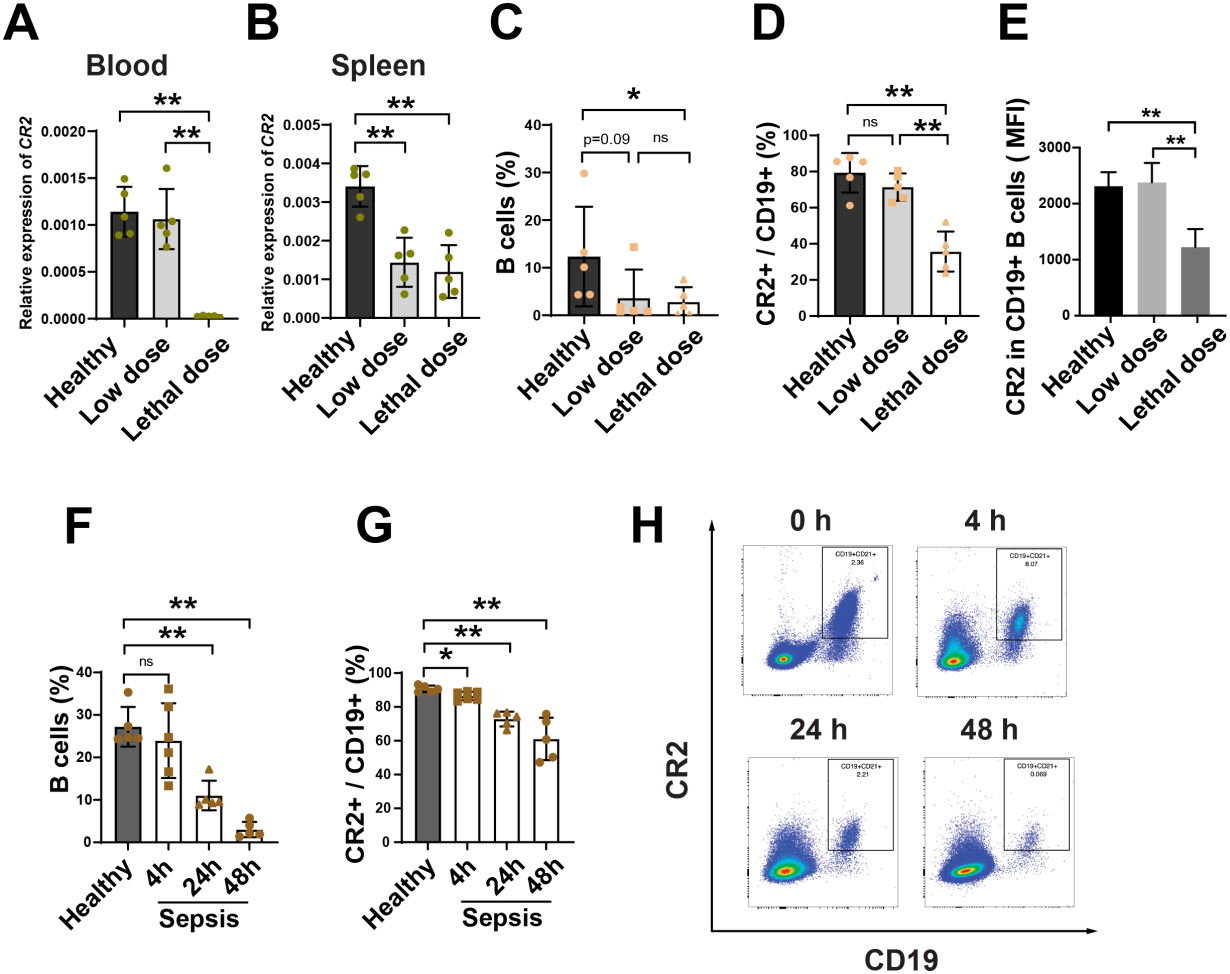
CR2 downregulation correlates with the infection severity in mice. **(A, B)** mRNA expression of *CR2* on Day 2 post-infection in the blood **(A)** and spleen **(B)** of NMRI mice intravenously infected with *S. aureus* Newman at a low dose (1 × 10^5^ CFU/mouse) or a lethal dose (1 × 10^8^ CFU/mouse), measured by RT-PCR. **(C–E)** Flow cytometry analysis of circulating B cells (% CD19^+^ of viable leukocytes) (**C**), CR2^+^ CD19^+^ B cells **(D)** and CR2 in CD19^+^ B cells (MFI). **(F, G)** Time-course analysis of B-cell frequencies **(F)** and CR2 expression **(G)** in mice infected with a lethal dose of (S) aureus, measured at 4, 24, and 48 h post-infection. **(H)** Representative flow cytometry plots illustrating the dynamic changes in CR2 expression in CD19^+^ B cells. Statistical analyses were performed using the Mann-Whitney *U*-test and the data are presented as the mean ± SD. *p < 0.05, **p < 0.01; n.s., not significant.

To further validate these findings, we conducted a dose-dependent infection study in mice using the *S. aureus* Newman strain. Mice were administered either a non-lethal dose (1 × 10^5^ CFU/mouse) or a lethal dose (1 × 10^8^ CFU/mouse) of the bacteria. The expression level of *CR2* mRNA on Day 2 post-infection was significantly downregulated in the blood samples from lethally infected mice, as compared with both the low-dose and control groups (**Figure 3A**). In the spleens, *CR2* expression was reduced at both doses, relative to the healthy controls (**Figure 3B**).

Flow cytometry confirmed a significant reduction in B-cell counts in the blood samples from lethally infected mice (**Figure 3C**), with a significant decline in CR2^+^ CD19^+^ B cells compared with both the low-dose infection group and controls. In addition, the mean fluorescence intensity (MFI) of CR2 on the remaining CR2-positive B cells was significantly reduced in lethal dose group compared to healthy and low dose group (**Figure 3E**). These findings indicate that the observed CR2 downregulation reflects both a loss of circulating B cells and reduced CR2 expression at the single-cell level. To assess the temporal dynamics, we conducted a time-course analysis at 4-, 24-, and 48-h post-infection. Total B-cell numbers were significantly reduced at 24 and 48 h (**Figure 3F**). CR2 expression was already diminished by 4 h and remained low throughout the experiment (**Figure 3G**). **Figure 3H** shows representative flow cytometry plots illustrating the progressive reduction of CR2 expression on CD19^+^ B cells. These findings demonstrate that CR2 expression, along with circulating B-cell numbers, change dynamically during *S. aureus* sepsis and are influenced by both bacterial dose and infection duration.

### CR2 downregulation is independent of C3, TLR2, and TNF signaling

**Figure 4:**
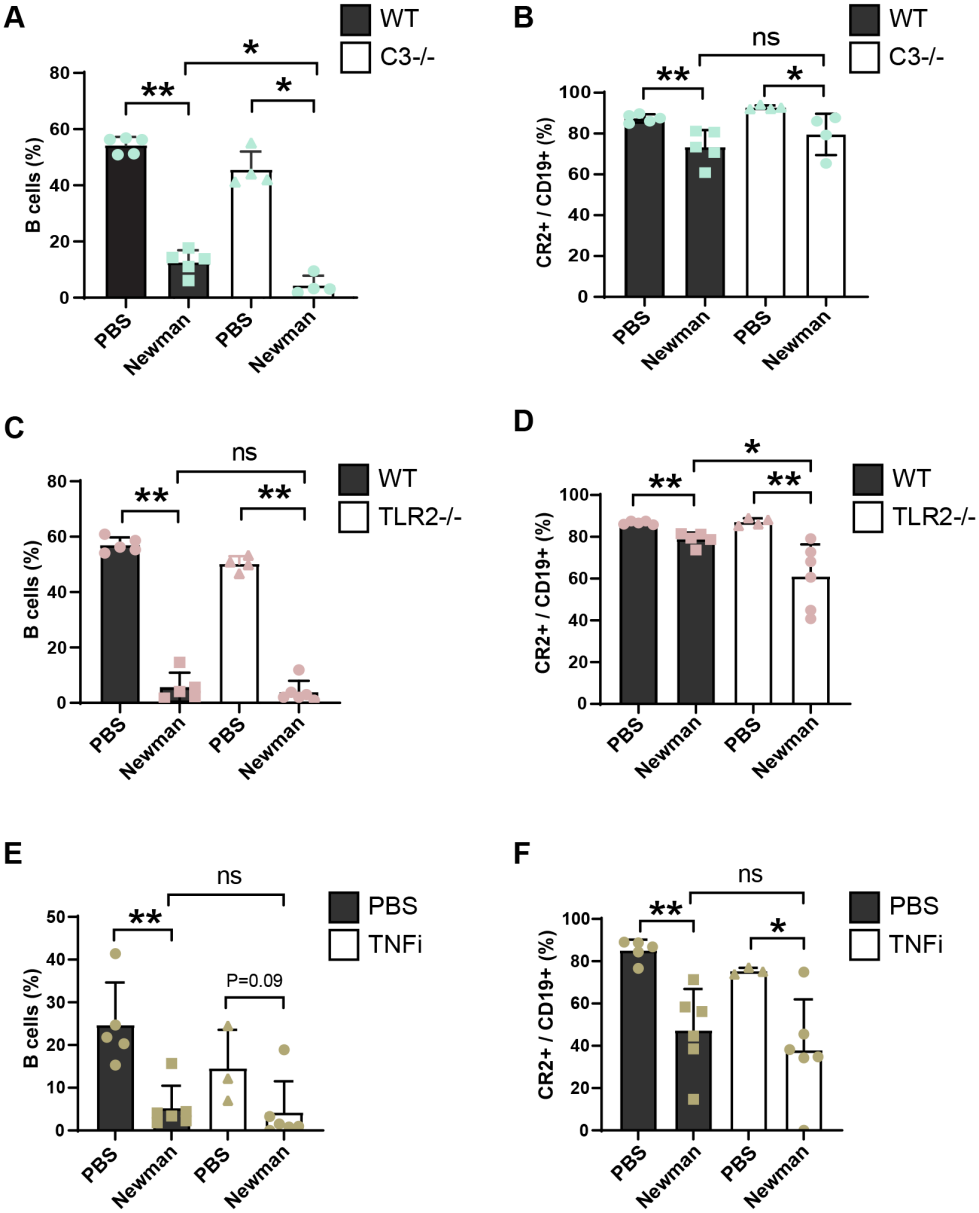
CR2 downregulation occurs independently of C3, TLR2, and TNF signaling in severe *S. aureus* sepsis. Frequencies of B cells (% CD19^+^ of viable single white blood cells) **(A, C, E)** and CR2^+^ CD19^+^ cells **(B, D, F)** on Day 2 post-infection in: C57BL/6 WT vs C3^-^/^-^ mice **(A, B)**; C57BL/6 WT vs TLR2^-^/^-^ mice **(C, D)**; and NMRI mice treated with TNF inhibitor (TNFi; etanercept, 500 μg/mouse, 24 h before infection and then daily) or PBS **(E, F)**. Mice were infected i.v. with *S. aureus* Newman at different doses (1.3 × 10^7^ to 1 × 10^8^ CFU/mouse) depending on host susceptibility. Statistical analyses were performed using the Mann-Whitney *U*-test; data are presented as mean ± SD. *p* < 0.05, *p < 0.01, n.s., not significant.

CR2 is known to interact with C3 fragments to facilitate B-cell activation (37). However, in *S. aureus*-infected C3^-^/^-^ mice, we observed similar reductions in B-cell numbers and CR2 expression as in infected WT mice (**Figures 4A, B**). This indicates that CR2 downregulation occurs independently of C3 in this setting.

Next, we examined the role of Toll-like receptor (TLR) 2, a key innate immune receptor for Gram-positive bacteria. Both WT and TLR2^-^/^-^ mice exhibited reduced B-cell counts and CR2^+^ B-cell frequencies (**Figures 4C, D**) in lethal sepsis. Notably, the frequency of CR2^+^ B cells were even lower in infected TLR2^-^/^-^ mice than in the infected WT mice (**Figure 4D**), suggesting that TLR2 may partially preserve CR2 expression during infection.

We also assessed the impacts of TNF inhibition on *S. aureus*-infected mice using etanercept treatment. TNF blockade did not restore the B-cell counts or *CR2* expression in cases of lethal sepsis (**Figures 4E, F**), suggesting that TNF is unlikely to be a major mediator of *CR2* downregulation in this model.

### *CR2* downregulation is associated with B-cell activation and redistribution to lymphoid organs

Given the reduction in the numbers of circulating B cells, we studied their fate during sepsis. For this, we infected mice with a lethal dose (1 × 10^8^ CFU/mouse) of *S. aureus* Newman. The expression levels of *Cd19, CR2, and Cd23* genes were significantly decreased in peripheral organs (kidney, liver, and lungs) 48 h post-infection (**Supplementary Figure 3**), suggesting that B cells do not accumulate in these tissues.

**Figure 5:**
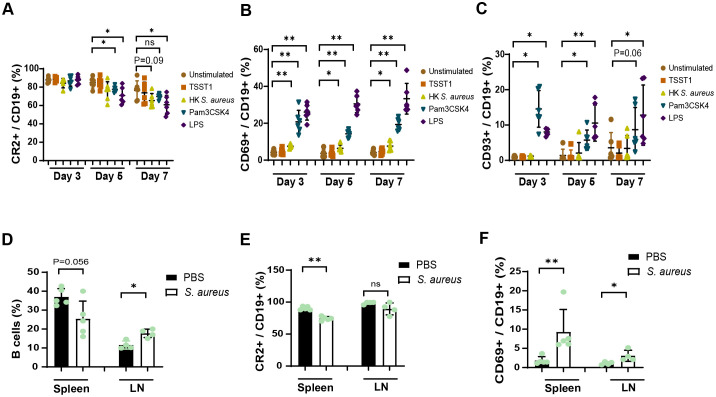
CR2 downregulation is driven by B-cell activation, differentiation into plasmablasts, and migration to secondary lymphoid organs upon *S. aureus* infection. Expression profiles of CR2 **(A)**, CD69 **(B)**, and CD93 **(C)** in NMRI mouse B splenocytes stimulated with TSST-1, heat-killed (HK) *S. aureus*, Pam3CSK4, and lipopolysaccharide (LPS). Frequencies of B cells (% CD19^+^ of viable single leukocytes) **(D)**, CR2^+^ CD19^+^ cells **(E)**, and activated B cells (% CD69^+^ CD19^+^). **(F)** in the spleens and lymph nodes of mice intravenously infected with *S. aureus* Newman (1 × 10^8^ CFU/mouse) or PBS-injected controls, analyzed 2 days post-injection. Statistical analyses were performed using the Mann-Whitney *U*-test and the data are presented as the mean ± SD. *p < 0.05, **p < 0.01; n.s., not significant.

To explore whether B cells undergo activation and differentiation in the *S. aureus*-infected animals, we stimulated murine splenic B cells with toxic shock syndrome toxin 1 (TSST-1), heat-killed *S. aureus*, staphylococcal lipopeptide (Pam3CSK4), and LPS. Compared with unstimulated B cells, CR2^+^ B cells were downregulated in the cell populations treated with Pam3CSK4 and LPS on Day 5, with LPS-induced downregulation persisting through Day 7 (**Figure 5A**). Marker of B-cell activation (CD69) was increased in all the cells treated with heat-killed *S. aureus*, Pam3CSK4 or LPS across all timepoints (**Figure 5B**). Plasmablast (CD93^+^ B cells) numbers were increased in Pam3CSK4-treated and LPS-treated samples on Days 3 and 5, with a further increase in numbers seen for LPS-treated cells by Day 7 (**Figure 5C**).

To determine if B cells redistribute to secondary lymphoid organs, we analyzed the spleens and lymph nodes of mice post-infection. Flow cytometry analysis revealed increased B-cell counts in the lymph nodes of *S. aureus* Newman-infected mice compared with PBS-treated controls (**Figure 5D**), while no significant differences in B-cell counts were observed in the spleens. CR2+ B cell frequencies were reduced in the spleens of the infected mice (**Figure 5E**), whereas both the spleen and lymph node cells exhibited increased activation, as compared with the controls (**Figure 5F**). These data suggest that B cells, in addition to differentiating into plasmablasts, may also redistribute to secondary lymphoid organs, particularly the lymph nodes, following infection.

### Staphylococcal virulence factors influence CR2 downregulation in *S. aureus* sepsis

**Figure 6:**
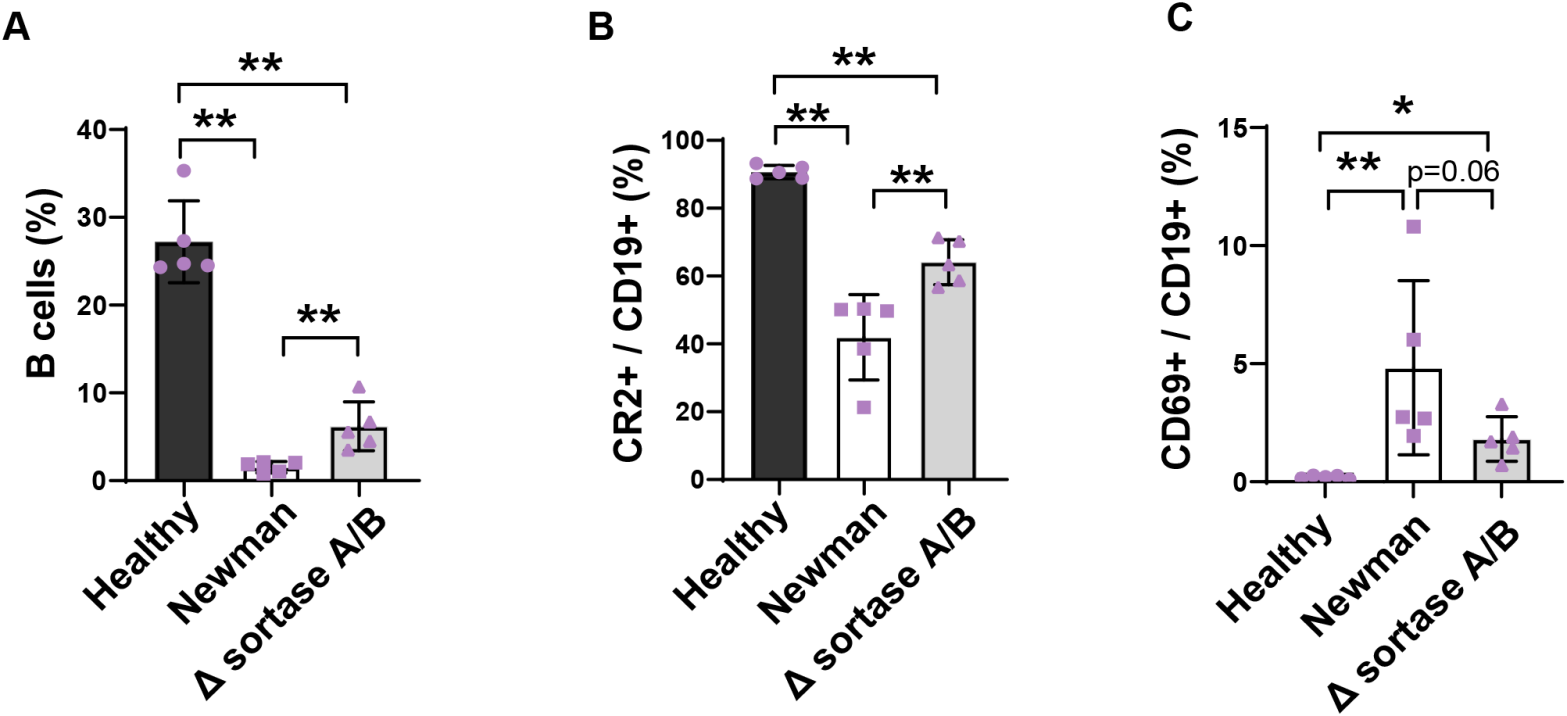
Contribution of staphylococcal virulence factors to CR2 downregulation during infection. Frequencies of B cells (% CD19^+^ of viable single leukocytes) **(A)**, CR2^+^ CD19^+^ cells **(B)**, and activated B cells (% CD69^+^ CD19^+^) **(C)** on Day 2 post-infection in NMRI mice intravenously infected with *S. aureus* Newman (WT) and *S. aureus* mutant (*Δsortase A/B*), each at a dose of 1 × 10^8^ CFU/mouse. Healthy mice injected with PBS served as the controls. Statistical analyses were performed using the Mann-Whitney *U*-test and the data are presented as the mean ± SD. *p < 0.05, **p < 0.01; n.s., not significant.

Sortases, which are key virulence factors of *S. aureus*, anchor surface proteins to the bacterial cell wall (38). To investigate whether bacterial virulence mechanisms influence CR2 regulation, we infected mice with either the *S. aureus* sortase A/B mutant (*Δsortase A/B*) or the parental Newman strain. Both strains gave reduced total B-cell counts, although the *Δsortase A/B*-infected mice had higher B-cell and CR2^+^ B-cell levels than the parental strain (**Figures 6A, B**). The CD69^+^ B-cell percentages were higher in both infection groups compared with the controls, with the parental strain inducing the highest activation levels (**Figure 6C**).

### Antibiotic treatment partially restores B-cell levels and CR2 expression

**Figure 7:**
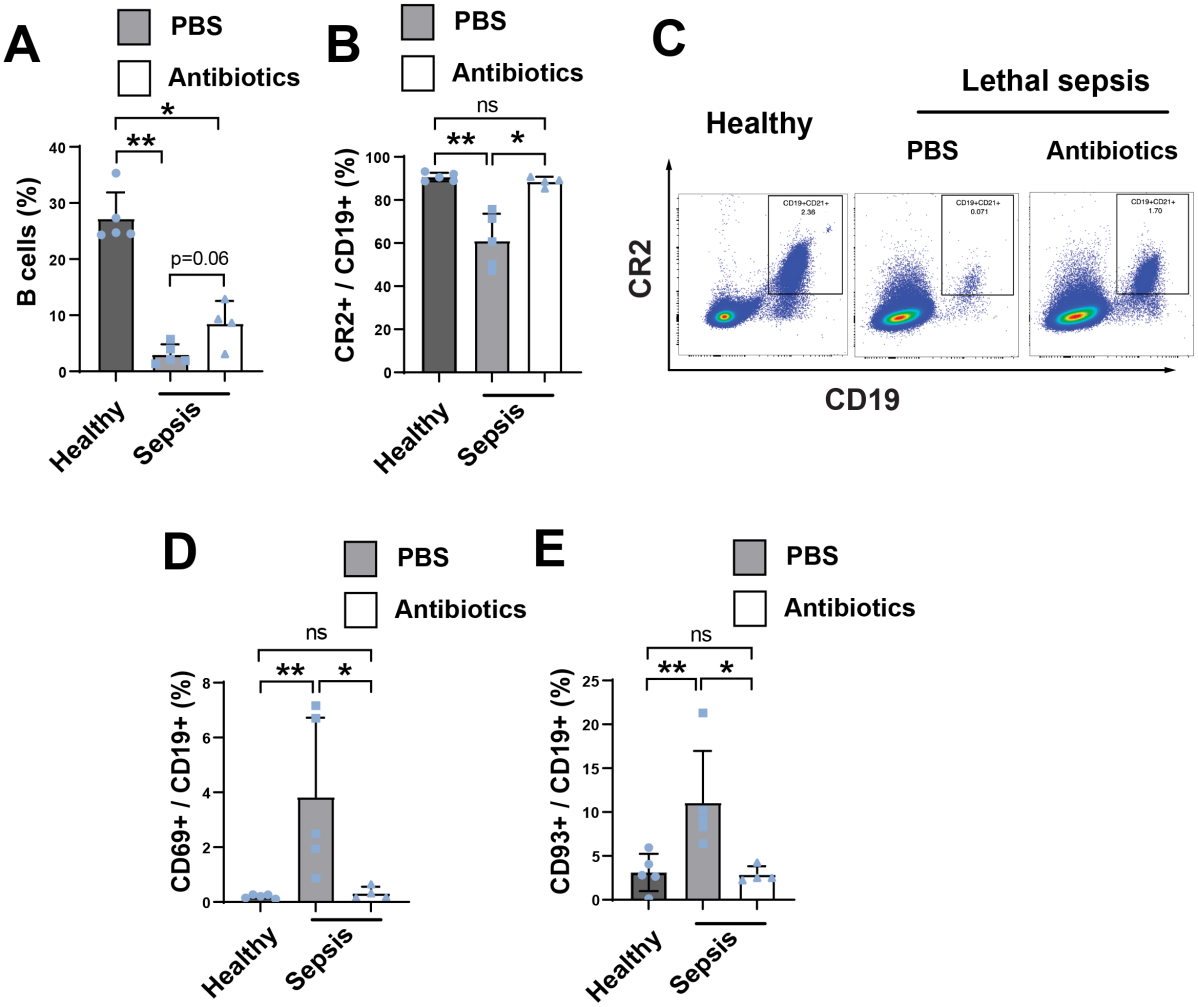
Antibiotic treatment restores B-cell numbers and CR2 expression during lethal *S. aureus* sepsis. Frequencies of B cells (% CD19^+^ of viable single leukocytes) **(A)**, CR2^+^ CD19^+^ cells **(B)**, activated B cells (% CD69^+^ CD19^+^) **(D)**, and CD93^+^ CD19^+^ cells on Day 2 post-infection in NMRI mice intravenously challenged with *S. aureus* Newman (1 ×10^8^ CFU/mouse). Mice were treated with cloxacillin (100 mg/mouse, twice daily) or an equal volume of PBS starting at 12 h post-infection until sacrifice at 48 h. Healthy mice were intravenously injected with PBS. **(C)** Representative flow cytometry plots demonstrating the restoration of CR2 expression in CD19^+^ cells. Statistical analyses were performed using the Mann-Whitney *U*-test and the data are represented as the mean ± SD. *p < 0.05, **p < 0.01; n.s., not significant.

Finally, we examined whether antibiotic therapy would restore CR2 expression. Mice infected with *S. aureus* Newman received cloxacillin starting 12 hours post-infection. Cloxacillin treatment increased circulating B-cell numbers, and significantly increased CR2^+^ B-cell frequencies (**Figures 7A, B**). Flow cytometry analyses confirmed restoration of the CR2^+^ B-cell numbers following antibiotic treatment (**Figure 7C**). Furthermore, cloxacillin treatment reduced CD69 expression (**Figure 7D**), and decreased the numbers of CD93^+^ plasmablasts (**Figure 7E**), indicating partial recovery of B-cell homeostasis following bacterial clearance.

## Discussion

Sepsis remains a major global cause of mortality, with few tools available for early risk stratification. In this study, we identified CR2 downregulation as a molecular feature associated with severe *S. aureus* infection. By integrating transcriptomics profiling in a murine sepsis model with analysis of patient samples, we show that reduced *CR2* expression is associated with disease severity and mortality. Notably, *CR2* expression was restored after antibiotic treatment, suggesting CR2 levels may reflect infection dynamics and host immune responses during treatment. We also demonstrated that *CR2* downregulation may reflect both reduced circulating B-cell numbers and changes in B-cell activation or differentiation states during severe *S. aureus* infection.

Our findings reveal a previously underappreciated aspect of B-cell dysfunction and differentiation in sepsis pathophysiology and suggest that CR2 may represent a link between innate and adaptive immune dysregulation during infection. Several biomarkers are currently used to predict sepsis outcomes, including routine clinical markers such as the levels of C-reactive protein (39, 40), and lactate (41), procalcitonin (42, 43), as well as the neutrophil-to-lymphocyte ratio (44), and soluble PD-L1 (40). However, given the heterogeneous and dynamic nature of sepsis, it is unlikely that a single biomarker can reliably predict outcomes (8). Instead, CR2 expression profiling may complement existing clinical, microbiological, immunological, and multi-omics tools to improve assessment of disease severity and host immune status. Unlike biomarkers such as CRP, procalcitonin, and lactate, which primarily reflect systemic inflammation or tissue hypoperfusion, CR2 may provide information regarding adaptive immune dysfunction and B-cell status, thereby capturing a distinct aspect of sepsis pathophysiology. Althrough further validation is largely required, these characteristics suggest that CR2 could potentially contribute additional prognostic information beyond conventional inflammatory markers.

Our data demonstrate that CR2 downregulation occurs independently of C3, TLR2, and TNF-α signaling. Interestingly, infected TLR2^-^/^-^ mice exhibited even lower frequencies of CR2^+^ B cells than infected wild-type mice. Although the precise mechanisms linking TLR2 signaling to CR2 expression remain unclear, these findings are consistent with a protective role of TLR2 during S. aureus sepsis. Indeed, S. aureus lipoproteins are among the most potent pro-inflammatory bacterial components and are recognized by TLR2, triggering robust chemokine production and recruitment of neutrophils to sites of infection (45, 46). Through these mechanisms, TLR2 serves as a key component of innate host defense against S. aureus and contributes to bacterial clearance and survival during sepsis (23). Therefore, the more pronounced reduction of CR2^+^ B cells observed in TLR2-deficient mice may reflect greater infection severity and a heightened degree of immune dysregulation in the absence of TLR2-mediated protective responses. Further studies are required to elucidate the pathways linking TLR2 signaling to the regulation of CR2 expression during severe infection.

We observed a marked reduction in peripheral B-cell numbers during severe sepsis, which likely is a major contributor to the downregulation of *CR2*. In contrast, CD23, another B-cell surface marker, failed to differentiate survivors from non-survivors among the patients with sepsis, suggesting that CR2 expression may capture aspects of B-cell dysregulation not reflected by total B-cell counts or CD23 expression alone. Severe sepsis is known to induce immunoparalysis, affecting both the innate and adaptive immune systems (8). Lymphopenia and adaptive immune dysfunction are well-established predictors of poor sepsis outcomes, with persistent lymphopenia correlating with the 28-day and 1-year mortality rates (47, 48). Early deficiencies in specific B-cell subsets, such as transitional and CD5^+^ B cells, have also been associated with poor prognosis (18).

Our data further suggest that peripheral B-cell depletion reflects both redistribution to lymphoid organs and differentiation into plasmablasts. Consistent with previous reports, critically ill patients with sepsis often exhibit lower absolute B-cell counts and increased apoptosis of class-switched and IgM memory cells (49). In our model, *CR2* downregulation is closely associated with B-cell activation and plasmablast differentiation, supporting the possibility that CR2 expression reflects dynamic changes in B-activation status during sepsis.

Interestingly, while B-cell numbers increased in the murine lymph nodes during infection, this phenomenon was not observed in the spleens. This discrepancy likely reflects differences in baseline cellular composition and compartment size. The murine spleen contains approximately 40%–60% B cells among its splenocytes (30–90 million B cells), whereas total circulating B cells in mice are estimated at 1–4 million. Thus, any blood-derived influx of B cells into the spleen would be relatively small and difficult to detect. In contrast, peripheral lymph nodes contain 1–3 million B cells per node, making blood-derived B-cell redistribution more readily measurable in these compartments.

Whether B-cell downregulation in sepsis merely reflects disease severity or contributes directly to pathogenesis remains unclear. Evidence from Rag1^-^/^-^ mice suggests the latter: these mice exhibit impaired early inflammation and poor survival in sepsis, which is reversed by B-cell reconstitution or transfer of wild-type serum, but not by T-cell transfer. This implicates a specific role for B cells in early host defense (50). Apoptosis is another contributor to immune dysregulation in sepsis, affecting both innate and adaptive immune cells through mediators such as steroids, TNF, nitric oxide, and FasL (51). In our model, B-cell apoptosis likely further contributes to B-cell loss and CR2 downregulation. It is noteworthy that adoptive transfer of apoptotic splenocytes has been shown to worsen survival in cecal ligation and puncture (CLP) sepsis models, an effect that is mediated by interferon-gamma (IFN-γ) (52). These findings underscore the immunosuppressive potential of apoptotic cells and raise the possibility that apoptotic B cells directly contribute to sepsis-associated immune suppression and mortality.

This study has several limitations. First, the patient cohort was small (N = 29), and the observed association may be influenced by age and other clinical confounders that could not be adequately addressed in this limited sample. In addition, information regarding major comorbidities was not systematically collected and was therefore not available for analysis. Second, the human analyses were based primarily on group-level comparisons rather than formal predictive modelling. Therefore, the association between CR2 expression and mortality should be interpreted cautiously. Larger prospective cohorts are required to determine whether CR2 provides independent prognostic information beyond established clinical markers. Third, the molecular mechanisms underlying *CR2* downregulation remain incompletely defined and warrant further investigation. Furthermore, we focused on *S. aureus* sepsis; whether similar *CR2* dynamics occur in polymicrobial or Gram negative bacterial infections remains unknown. Finally, we measured CR2 at the mRNA level, which may limit clinical applicability. Future studies should evaluate whether CR2 protein levels can be measured using rapid and clinically practical assays.

**Figure 8:**
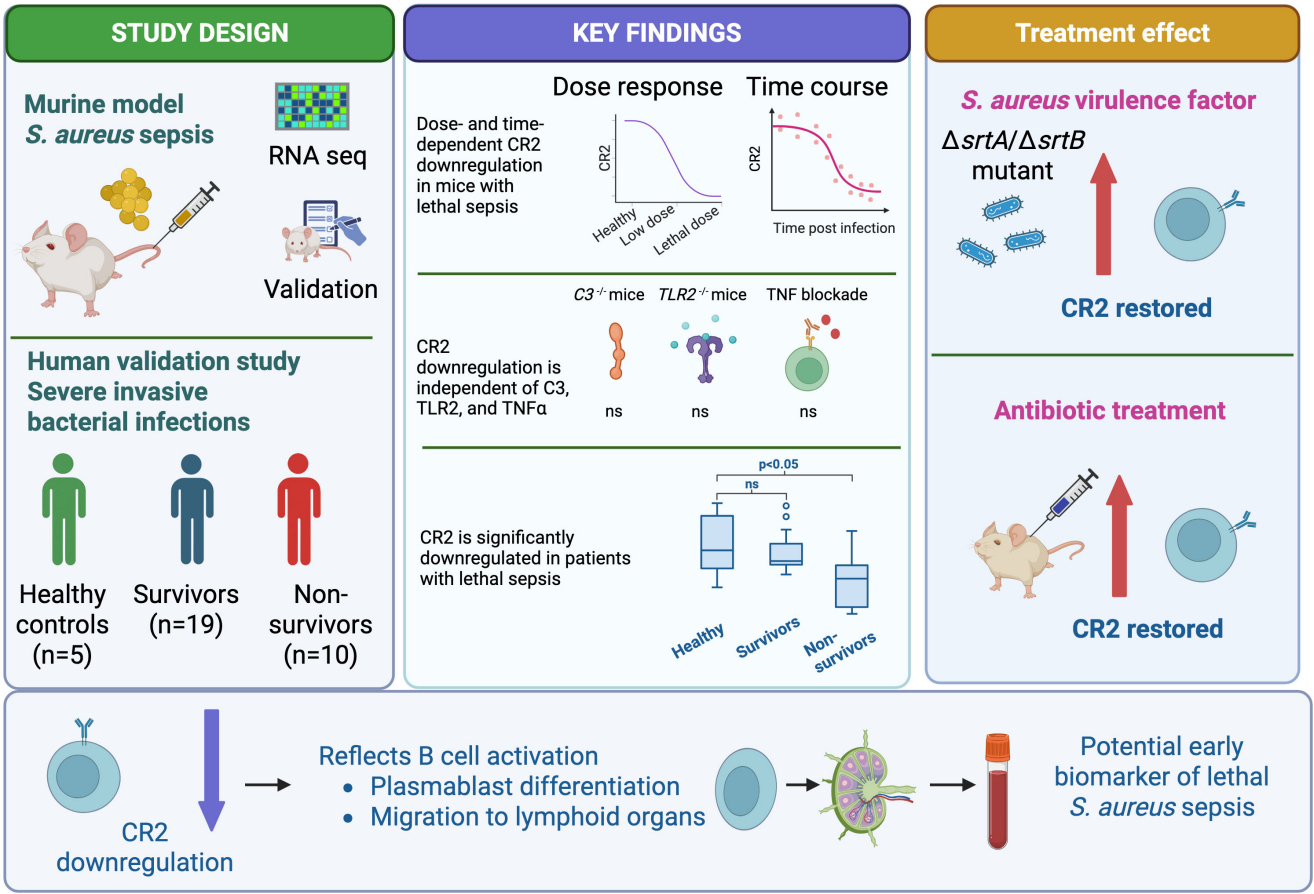
Graphical abstract summarizing the association between CR2 downregulation and severe *Staphylococcus aureus* sepsis. CR2 expression is reduced in both experimental mouse models and patients with sepsis and is associated with disease severity and mortality. The findings suggest that CR2 downregulation reflects alterations in B-cell activation and differentiation during infection and may serve as a potential biomarker of host immune dysregulation in severe S. aureus sepsis.

The main findings of the present study are summarized in the graphical abstract (**Figure 8**). In summary, we demonstrate that *CR2* downregulation is associated with severe infection and mortality in *S. aureus* sepsis and reflects alterations in B-cell activation, and differentiation during infection. Using both human clinical samples and mouse models, we show that *CR2* downregulation is an early and consistent feature of severe infection and can be influenced by both bacterial virulence factors and antibiotic treatment. Our findings provide new insights into B-cell immunopathology during sepsis and suggest that CR2 might represent a candidate biomarker of host immune dysregulation that warrants further investigation in the larger clinical studies.

## Data Availability

The datasets presented in this study can be found in online repositories. The names of the repository/repositories and accession number(s) can be found at: https://www.ncbi.nlm.nih.gov/, GSE222530.
