# Supplementary material for: Complement receptor 2 downregulation is associated with mortality in Staphylococcus aureus sepsis in both mice and humans
Source: Front Immunol. 2026 Jul 9;17:1724049. doi: 10.3389/fimmu.2026.1724049 (PMC13391265; doi:10.3389/fimmu.2026.1724049)
Supplement: Supplementary file 1 [file DataSheet1.pdf]

***Complement receptor 2* downregulation is associated with mortality in *Staphylococcus aureus sepsis* in both mice and humans**

Pradeep Kumar Kopparapu[1], Meghshree Deshmukh[1#], Santhilal Subhash[2,3#], Majd Mohammad[1], Zhicheng Hu[1,4], Anders Jarneborn[1,5], Muhammad Arif[6,7], Marcela Pekna[8], Lars Ljungström[9], Gunnar C Jacobsson[9], Ola Grimsholm[1,10], Tao Jin[1,5*]

[1]Department of Rheumatology and Inflammation Research, Institute of Medicine, The Sahlgrenska Academy, University of Gothenburg, Gothenburg, Sweden.

[2]Cold Spring Harbor Laboratory, Cold Spring Harbor, NY, USA.

[3] Department of Biosciences and Bioengineering, Indian Institute of Technology Jammu, Jammu, Jammu and Kashmir, India.

[4]Center for Clinical Laboratories, The Affiliated Hospital of Guizhou Medical University, Guiyang 550001, China.

[5]Department of Rheumatology, Sahlgrenska University Hospital, Gothenburg, Sweden.

[6]Department of Molecular and Clinical Medicine, Institute of Medicine, The Sahlgrenska Academy, University of Gothenburg, Gothenburg, Sweden.

[7]Science for Life Laboratory, University of Gothenburg, Gothenburg, Sweden.

[8]Center for Brain Repair and Rehabilitation, Department of Clinical Neuroscience and Rehabilitation, Institute of Neuroscience and Physiology, The Sahlgrenska Academy, University of Gothenburg, Gothenburg, Sweden.

[9]Department of Infectious Diseases, Skaraborg Hospital, Skövde, Sweden

[10]Institute of Pathophysiology and Allergy Research, Center for Pathophysiology, Infectiology and Immunology, Medical University of Vienna, Austria

[#]Contributed equally

*Corresponding author:

Tao Jin, MD, PhD, Associate Professor

Department of Rheumatology & Inflammation Research, Institute of Medicine,

Guldhedsgatan 10A,

University of Gothenburg-41346, Sweden

Email: tao.jin@rheuma.gu.se

Running title: *CR2* downregulation in severe sepsis

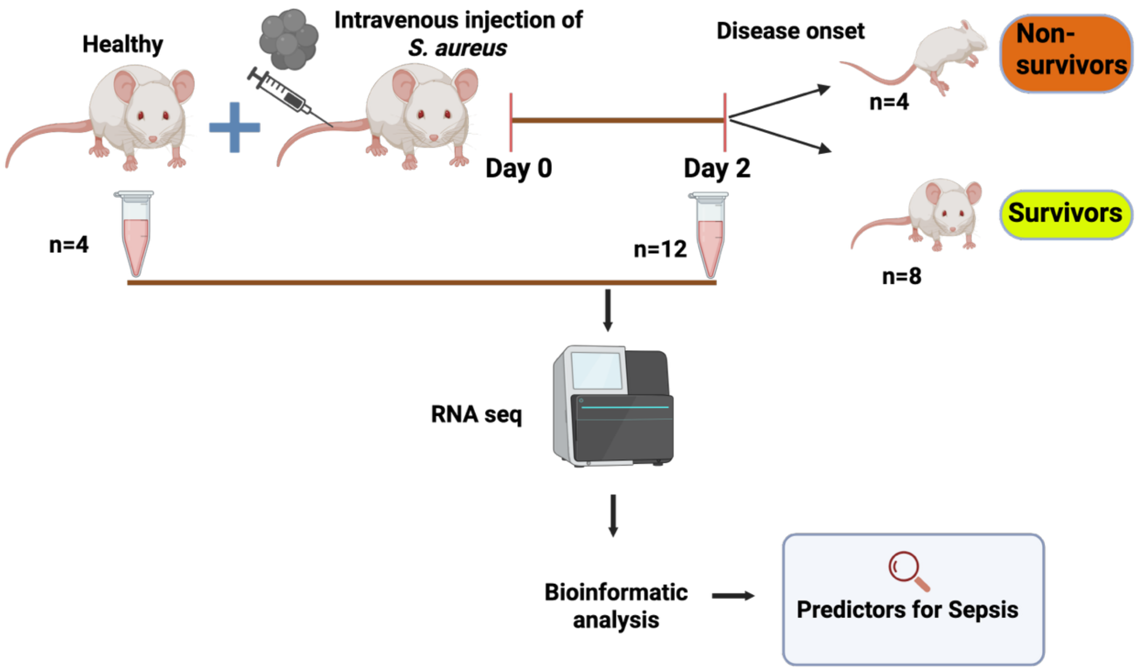

Supplementary Figure 1: Schematic overview of the experimental design. NMRI mice (n = 12) were injected i.v. with 200 µL of the Newman WT strain (2 × 10^7 CFU per mouse). To capture potential predictive biomarkers before disease onset, blood samples were collected on Day 2 post-infection. Mortality was monitored daily until Day 10. Blood samples from Days 0 and 2 were used for RNA sequencing. Based on the mortality outcomes, the mice were stratified into two groups (survivors and non-survivors), and the RNA sequencing data were analyzed to identify gene expression patterns that distinguished these groups.

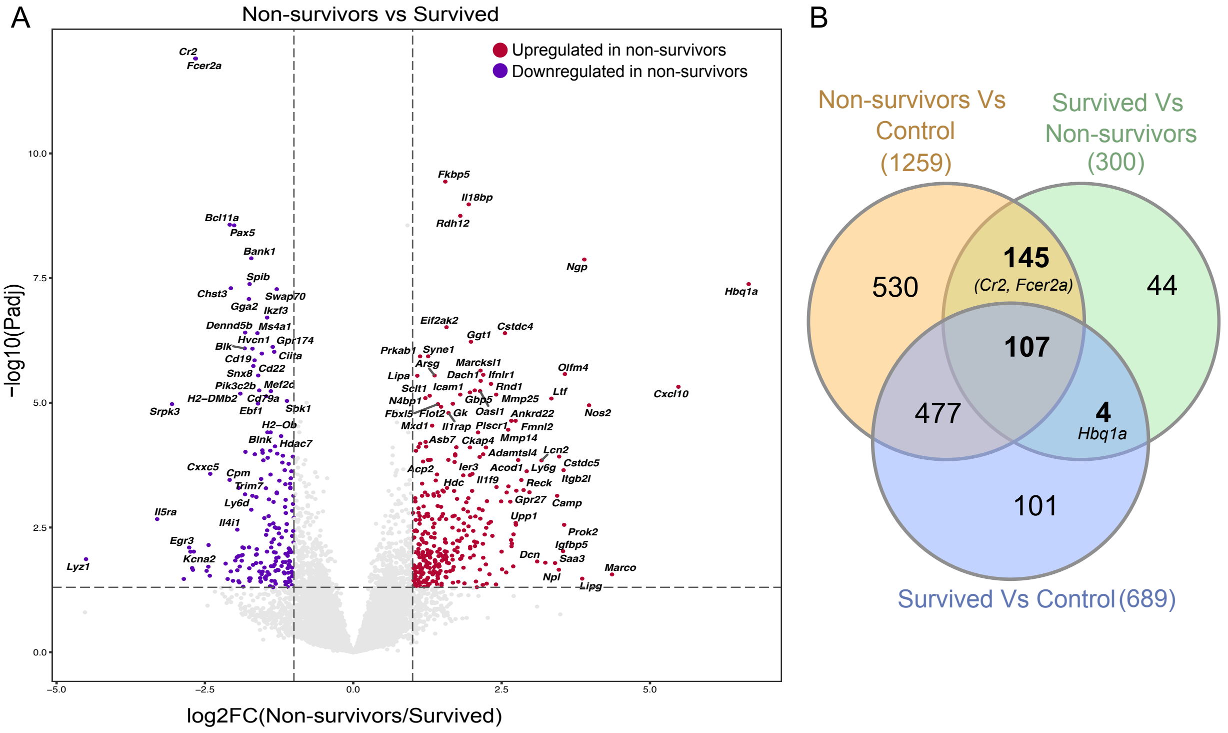

Supplementary Figure 2: Direct transcriptomics analysis of surviving and non-surviving mice following *S. aureus* infection. NMRI mice (n = 12) were infected intravenously with the *Staphylococcus aureus* (*S. aureus*) Newman strain (2 $\times 10^7$ CFU/mouse). Blood samples were collected at baseline (Day 0) and on Day 2 post-infection, prior to the onset of clinical sepsis. Mice were monitored twice daily until Day 10, during which four mice died (non-survivors) and eight survived (survivors). RNA sequencing was performed on blood samples from non-survivors (NS, n = 4) and survivors (S, n = 8). Bioinformatic analyses were performed to compare transcriptional profiles between non-survivors and survivors. **(A)** Volcano plots showing differentially expressed genes between Day 2 samples from non-survivors or survivors (S). The horizontal dotted line indicate a false discovery rate (FDR) cut-off of 0.05, and the vertical dotted lines indicate a $\log_2$-fold change cut-off of ±1. The red and blue colors indicate upregulated and downregulated genes, respectively. **(B)** The Venn diagram depicts the various sets of genes regulated on day 2 (Survivors

and non survivors) relative to healthy controls. A total of 1,259 genes were differentially expressed in non-survivors compared with controls, 689 genes in survivors compared with controls, and 300 genes between survivors and non-survivors.

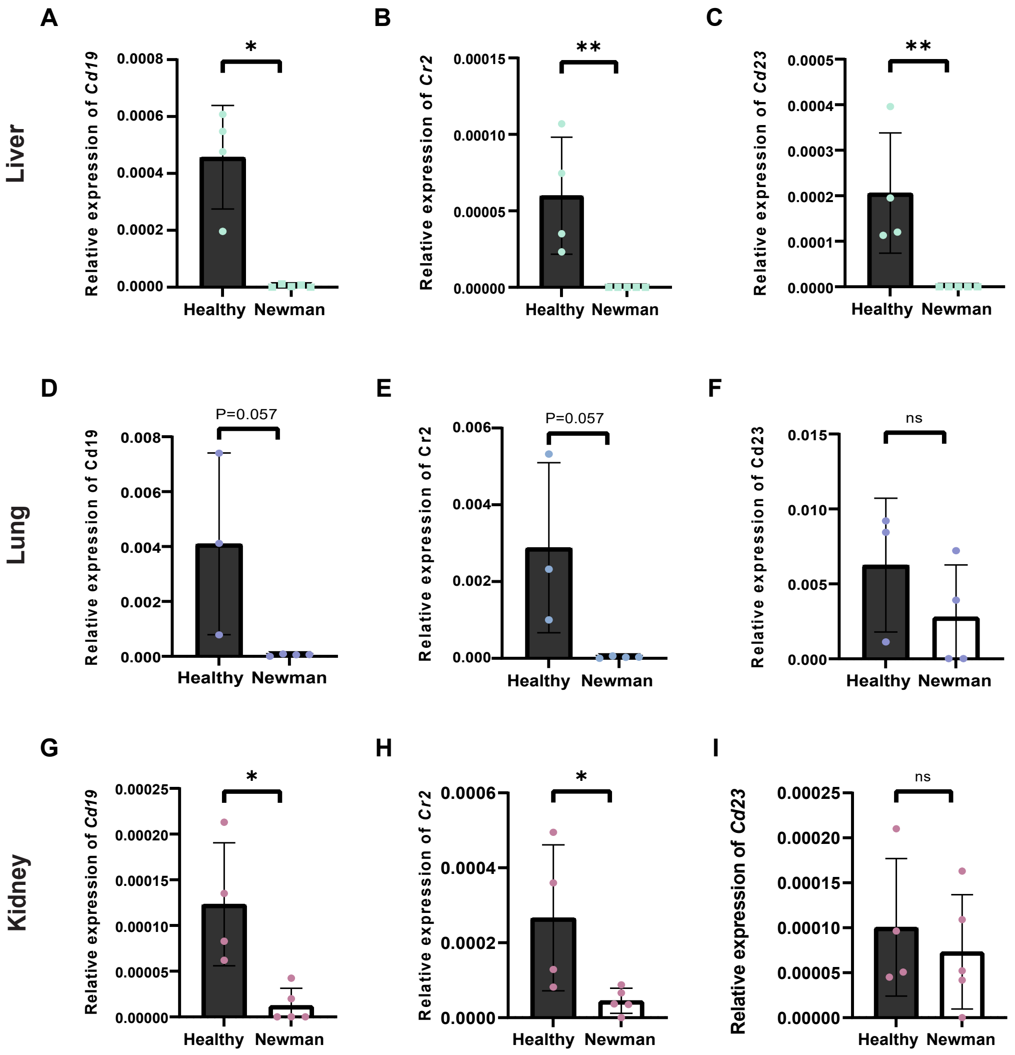

Supplementary Figure 3: Gene expression levels in the organs of healthy mice and mice with *Staphylococcus aureus* sepsis. NMRI mice were infected i.v. with the *S. aureus* Newman strain (1 ×10^8 CFU/mouse; n=4) or injected with an equal volume of PBS (n=5). Kidneys, lungs, and livers were harvested on Day 2 post-infection. Shown are the relative expression levels in healthy mice and *S. aureus* Newman strain-infected mice of: *Cd19* (**A**), *Cr2* (**B**) and *Cd23* (**C**) in the livers; *Cd19* (**D**), *Cr2* (**E**) and *Cd23* (**F**) in the lungs;and *Cd19* (**G**), *Cr2* (**H**) and *Cd23* (**I**) in the kidneys.

Statistical analyses were performed using the Mann-Whitney *U*-test, and the data are presented as the mean ± SD. *$p < 0.05$, **$p < 0.01$; n.s = not significant.

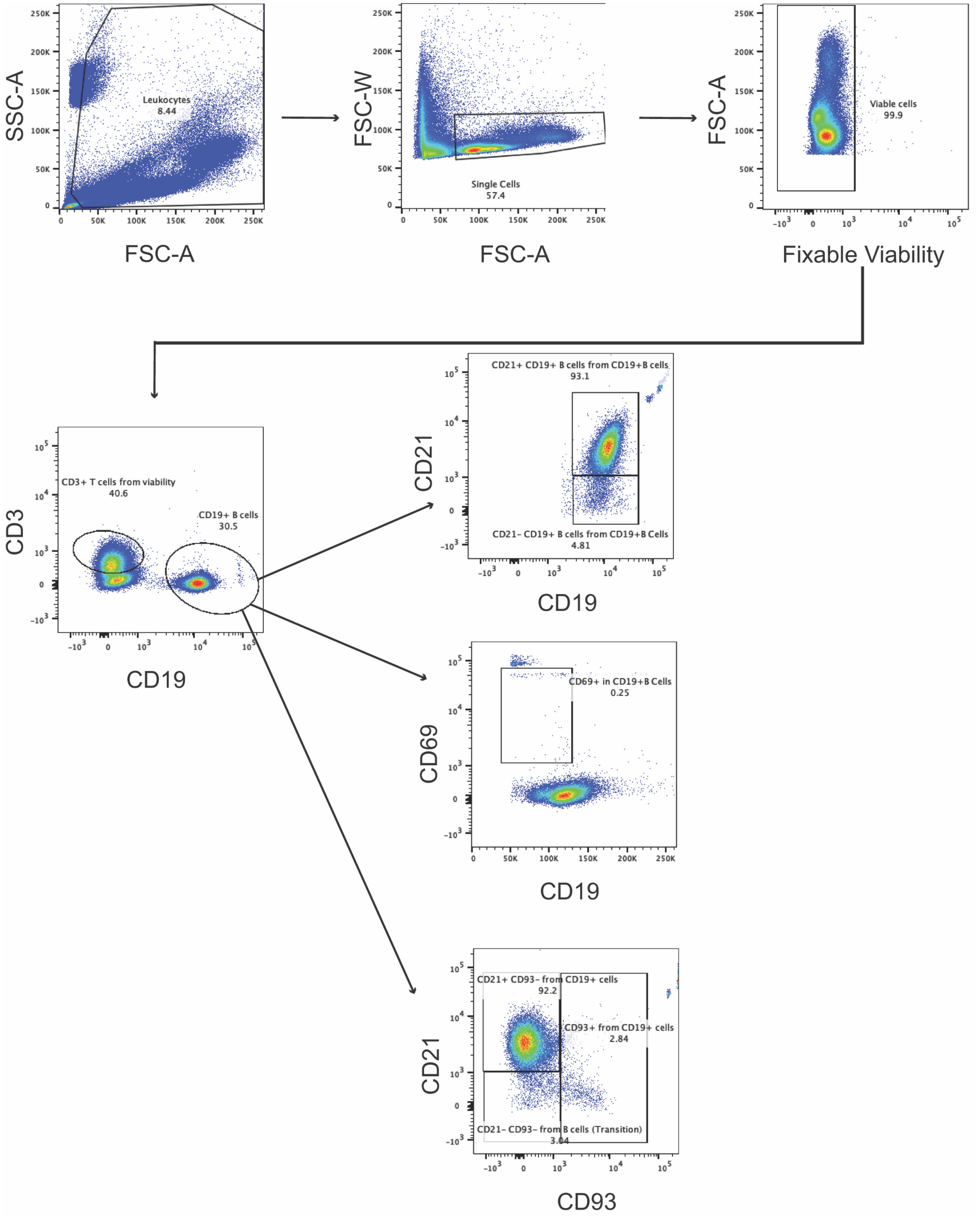

Supplementary Figure 4. Representative flow-cytometry gating strategy for analysis of CR2 positive B cells, CD69 expression, and CD93 expression in peripheral blood. Mouse blood

cells were collected into EDTA tubes. Erythrocytes were lysed using RBC Lysis Buffer (eBioscience; Invitrogen, Waltham, MA, USA). Cells were stained with the following fluorochrome-conjugated antibodies: phycoerythrin (PE)-conjugated anti-CD19 (BioLegend, San Diego, CA, USA); APC-Cyanine7–conjugated anti-CD21/CR2 (BioLegend); fluorescein isothiocyanate (FITC)-conjugated anti-CD69 (Invitrogen); and allophycocyanin (APC)-conjugated anti-CD93 (Invitrogen). Fixable Viability Dye e506 (Invitrogen) was included to discriminate live cells from dead cells. Leukocytes were first identified based on FSC-A and SSC-A, followed by exclusion of doublets using FSC-A versus FSC-W. Dead cells were excluded using Fixable Viability Dye e506, and subsequent analyses were performed on viable cells. $CD19^+$ B cells were then gated from the viable-cell population. Within the $CD19^+$ B-cell gate, $CD21/CR2^+$ cells were identified using CD21 staining. CD69 expression was assessed among $CD19^+$ B cells, and CD93 expression was evaluated together with CD21/CR2 to identify distinct B-cell subpopulations based on their maturation phenotype. Representative plots are shown from a healthy control mouse. Samples were acquired on a BD FACSLyric flow cytometer (BD Biosciences, Franklin Lakes, NJ, USA), and data were analyzed using the FlowJo ver. 10.8 software (BD Biosciences).

Supplementary Table 1. Relative expression of 16 human orthologs (matching the top differentially expressed mouse genes) in sepsis patients (survivors vs. non-survivors) and healthy controls.

Gene	Healthy (N=5) (median)	Patients (N=29) (median)	P-value	Survivors (N=19) (median)	Non-survivors (N=10) (median
CR2	0.0004360	0.0001210	**0.0295**	0.0001830	5.610e-005
CD23	0.0001372	0.0001959	0.9624	0.0002587, n=19	0.0001511
STEAP4	1.660e-005	9.700e-006	0.3351	1.030e-005	8.665e-006
S100A9	0.4234	0.5488	0.5706	0.5488	0.4492
LTF	0.0001410	0.0002550	**0.0647**	0.0002450	0.0004695
PAX5	0.0002130	0.0001540	>0.9999	0.0001540	0.0001605
ANGPTL1	0.0001380	0.0002040	0.2321	0.0002040	0.0001860
CXCL10	6.070e-006	9.570e-006	0.6340	8.300e-006	1.775e-005
OLFM4	0.0001575	0.0002190	0.9714	0.0002190	0.0002410
CD33	0.01751	0.05012	0.2321	0.04386	0.05289
CAMP	0.01745	0.02692	0.5706	0.02323	0.02810
IL1R2	0.01145	0.04621	**0.0392**	0.03914	0.07770
S100A8	0.0002659	0.0005931	**0.0334**	0.0005931	0.0006383
ENTPD3	0.0003890	0.0001730	0.1350	0.0001780	0.0001262
MMP25	0.002453	0.003748	0.3416	0.003748	0.003510
SRPK3	0.04886	0.06159	>0.9999	0.03179	0.08297

Supplementary Table 2. Primers used for RT-PCR analysis of gene expression in this study.

Gene	Organism	Forward Primer (5′-3′)	Reverse Primer (5′-3′)
Cd19	Mouse	GCTCTGAATTCTATGAGAACG	CATAAGACTCAGCATTGGAG
Cd23	Mouse	GGGTTATAGCAACTGGAATC	CAGATATCTCACATGTTGCC
Cr2	Mouse	GAGGACACTATACATTGCAC	CTTAACTGGAACCCTTCATC
Actb	Mouse	GATGTATGAAGGCTTTGGTC	TGTGCACTTTTATTGGTCTC
CR2	Human	ATCCTAAGAGGCCGAATGGT	CTTTTGCCCATTGAGGATGT
CD23	Human	GACACCTGCAACTCCATCCT	GTATGCCTGTGACGACATGG
STEAP 4	Human	GCTGAGGAGGTGCTAAATGG	GCCCTTCAMTCTCAGATGG
S100A8	Human	CCTGTAGACGGCATGGAAAT	CCAGGAGTTCCTCATTCTGG
S100A9	Human	TCATCAACACCTTCCACCAA	GTGTCCAGGTCCTCCATGAT
LTF	Human	CAAGTTCTGGATGGCAGTGA	GGTGCTGGAAAAGTTTGGAA
PAX5	Human	TCGTCTCTCTTGCGCTTGTT	CATCTTCACCACCACAGAGC
ANGPTL1	Human	TTGGTCCATTGCTGTTTTCA	TATAGACTGCGCCTGGGAAC
CXCL10	Human	CAAAATTGGCTTGCAGGAAT	GAATCGAAGGCCATCAAGAA
OLFM4	Human	CAGAGTGGAACGCTTGGAAT	CCTTGATCAGCTCGAAGTCC
CD33	Human	ACTCACTCCTCGGTGCTCAT	TTGTTGGGTTCTGTGGAACA
CAMP	Human	GCCAAAGCCTGTGAGCTTC	GGTCACTGTCCCCATACACC
IL1R2	Human	CCTTGTCAACCTCTGGGGTA	ACAGCGGTAATAGCCAGCAT
ENTPD3	Human	AAGATGTCCCCAGAGCCTTT	TGCTGTTTCATTTTGCAACC
MMP25	Human	CCTGACATGGAGGGTACGTT	GGCAAAGTCGATGAGGATGT
SRPK3	Human	GCCCTCAAAGTGGTGAAGAG	CTCTCTTTTGGGGTCACTGG
ACTB	Human	CACCATTGGCAATGAGCGGTTC	AGGTCTTTGCGGATGTCCACGT

Supplementary Table 3. Demographic, clinical, and microbiological data for 29 patients with invasive bacterial infections, showing survival outcomes.

Patient	Age	Gender	Clinical diagnosis and pathogens	Survivor / Non-survivor
**1**	84	M	*Staphylococcus aureus* bacteremia	Non-survivor
**2**	76	M	*Staphylococcus aureus* bacteremia	Non-survivor
**3**	49	M	Bursitis, *Staphylococcus aureus*	Survivor
**4**	86	F	Septic shock, *Klebsiella pneumoniae*	Survivor
**5**	71	F	Septic arthritis hand, group C streptococci	Survivor
**6**	86	M	*Staphylococcus aureus* bacteremia	Non-survivor
**7**	93	M	*Staphylococcus aureus* bacteremia	Non-survivor
**8**	77	F	Septic arthritis of shoulder, *Staphylococcus aureus*	Survivor
**9**	43	F	Septic shock, *Klebsiella pneumoniae*	Survivor
**10**	75	M	Spondylodiscitis, *Staphylococcal aureus* bacteremia	Survivor
**11**	57	M	Septic shock, *Streptococcus pyogenes*	Survivor
**12**	87	F	Prosthesis arthritis of knee, *Staphylococcus aureus* bacteremia	Survivor
**13**	75	M	*Staphylococcus aureus* bacteremia	Non-survivor
**14**	82	M	Prosthesis arthritis of hip, Bacteroides bacteremia	Survivor
**15**	96	F	*Staphylococcus aureus* bacteremia	Non-survivor
**16**	55	F	Pneumococcal bacteremia	Non-survivor
**17**	67	F	Septic arthritis shoulder, *Staphylococcus aureus* bacteremia	Survivor
**18**	65	F	Septic shock, *Pseudomonas aeruginosa*	Survivor
**19**	75	F	Septic arthritis foot, *Staphylococcus aureus* in joint	Survivor
**20**	49	M	Septic shock pneumococci	Survivor
**21**	85	M	*Staphylococcus aureus* bacteremia	Non-survivor
**22**	60	F	Septic shock, *Escherichia coli*	Survivor
**23**	84	F	Prosthesis arthritis of hip, *Staphylococcal aureus* bacteremia	Non-survivor
**24**	91	F	Septic shock, *Escherichia coli* bacteremia	Survivor
**25**	75	M	Bursitis, *Staphylococcus aureus* bacteremia	Survivor
**26**	49	M	Septic arthritis of finger, *Staphylococcal aureus*	Survivor
**27**	45	F	Septic shock, *Escherichia coli* bacteremia	Survivor
**28**	65	M	*Staphylococcus aureus* bacteremia	Non-survivor
**29**	72	M	Septic shock, septic arthritis of shoulder, *Staphylococcus aureus* bacteremia	Survivor
